# MicroRNA-21 as a Regulator of Cancer Stem Cell Properties in Oral Cancer

**DOI:** 10.3390/cells14020091

**Published:** 2025-01-10

**Authors:** Milica Jaksic Karisik, Milos Lazarevic, Dijana Mitic, Maja Milosevic Markovic, Nicole Riberti, Drago Jelovac, Jelena Milasin

**Affiliations:** 1 Department of Human Genetics, School of Dental Medicine, University of Belgrade, Dr. Subotica 8, 11000 Belgrade, Serbia; milica.jaksic@stomf.bg.ac.rs (M.J.K.); milos.lazarevic@stomf.bg.ac.rs (M.L.); dijana.trisic@stomf.bg.ac.rs (D.M.); maja.milosevic@stomf.bg.ac.rs (M.M.M.); 2 Department of Neuroscience, Imaging and Clinical Sciences, University of Chieti-Pescara, 66100 Chieti, Italy; nicole.riberti@unich.it; 3 Clinic for Maxillofacial Surgery, School of Dental Medicine, University of Belgrade, Dr. Subotica 8, 11000 Belgrade, Serbia; drago.jelovac@stomf.bg.ac.rs

**Keywords:** oral cancer, miRNA-21, cancer stem cells, apoptosis, miRNA-21 inhibition

## Abstract

Oral squamous cell carcinoma (OSCC) is a highly aggressive malignancy with poor prognosis, mainly due to the presence of cancer stem cells (CSCs), a small subpopulation of cells that contribute to therapy resistance and tumor progression. The principal objective of this study was to investigate the role of miRNA-21 in the maintenance of cancer cell stemness and the possibility of altering it. The CD44 antigen was used as a marker for CSC isolation from oral cancer cell cultures. CD44+ and CD44− populations were sorted via magnetic separation. miRNA-21 inhibition was performed in CD44+ cells via transfection. CD44+ cells possessed a significantly higher migration and invasion potential compared to CD44− cells, higher levels of miRNA-21 (*p* = 0.004) and β-catenin (*p* = 0.005), and lower levels of BAX (*p* = 0.015). miRNA-21 inhibition in CD44+ cells reduced migration, invasion, and colony formation while increasing apoptosis. Stemness markers were significantly downregulated following miRNA-21 inhibition: *OCT4* (*p* = 0.013), *SOX2* (*p* = 0.008), and *NANOG* (*p* = 0.0001), as well as β-catenin gene (*CTNNB1)* (*p* < 0.05), an important member of WNT signaling pathway. Apoptotic activity was enhanced, with a significant downregulation of the antiapoptotic Bcl-2 (*p* = 0.008) gene. In conclusion, miRNA-21 plays a critical role in the regulation of oral cancer CD44+ cells properties. Targeting and inhibiting miRNA-21 in CD44+ cells could represent a promising novel strategy in OSCC treatment.

## 1. Introduction

Cancer represents the principal cause of death for individuals under the age of 70, and the global burden is increasing. In 2020, there were 19.3 million new cancer cases and approximately 10 million deaths attributed to the disease worldwide, with projections indicating a 47% rise in cancer cases over the next two decades. Among these, oral cancer is particularly concerning, especially in regions like South-Central Asia where it is an important cause of mortality [1]. Alarmingly, almost half of all oral cancer cases are diagnosed at advanced stages (III and IV), where the five-year survival rate plummets to 27.8%. Also, even tumor-free margins have been shown to harbor a high incidence of genetic lesions usually associated with advanced oral cancer [2]. However, when detected early, survival rates improve considerably to 51.6% [3].

One of the major challenges in cancer treatment is drug resistance, particularly due to the presence of cancer stem cells (CSCs). These CSCs, characterized by their capability to self-renew and drive tumor progression, are highly resistant to both chemotherapy and radiation therapy, often leading to tumor recurrence and metastasis [4]. Specific markers such as *OCT4, SOX2, NANOG, ALDH1*, and *CD44* are commonly used to identify CSCs, with CD44 glycoprotein being the most widely recognized due to its correlation with higher tumor grades and later clinical stages [5].

MicroRNA (miRNA) are small non-coding RNAs that regulate gene expression by influencing mRNA stability and translation, which in turn may affect key cancer-related processes like cell growth and apoptosis [6]. Abnormal miRNA expression can lead to cancer progression, with both oncogenic and tumor-suppressive miRNAs contributing to this process [7]. Certain miRNAs, such as miRNA-21, miRNA-31, and miRNA-125b, have been identified as playing critical roles in tumor formation and metastasis [8]. Moreover, miRNAs are involved in maintaining cancer stem cell (CSC) properties and regulating crucial signaling pathways such as Wnt/β-catenin, PI3K/Akt, and NF-κB, which are important for cancer survival and drug resistance [9]. Targeting dysregulated miRNAs holds promise for overcoming drug resistance and improving therapeutic outcomes.

MiRNA-21 is recognized as an “oncomiR” due to its widespread deregulation in numerous cancer types. It was first identified as an inhibitor of apoptosis across various cell lines and is now considered one of the most prominently overexpressed microRNAs in cancers such as lung, breast, stomach, prostate, colon, and pancreas. In particular, large-scale studies have shown that miRNA-21 is consistently overexpressed in six solid cancer types (breast, colon, lung, stomach, prostate, and pancreatic cancers), which highlights its importance as an oncogenic miRNA [10,11,12].

Additionally, miRNA-21 has been proposed as a potential biomarker for malignancies, being detectable in various body fluids such as blood, sputum, cerebrospinal fluid, and feces, making it useful for non-invasive cancer diagnostics [13]. In pancreatic ductal adenocarcinoma (PDAC), miRNA-21 has been linked to the regulation of key stemness markers which contribute to cancer cell survival, invasion, and chemoresistance, such as *CD44*, *CD133* and *CXCR4* [14,15]. The knockout of miRNA-21 in PDAC cells has been shown to significantly reduce these factors, highlighting its potential as a therapeutic target [14,16]. Overexpression of miRNA-21 has also been associated with glioblastoma resistance to temozolomide, the standard first-line treatment, by impacting the balance between the pro-apoptotic Bax protein and the anti-apoptotic Bcl-2, as well as by influencing Caspase 3 activity [17]. miRNAs, including miRNA-21, are stably detectable in plasma and serum, making them promising molecular biomarkers for minimally invasive cancer diagnosis and prognosis. High expression of miRNA-21 in serum and tissues has been correlated with larger tumor size, metastasis, and poor outcome [18]. Recent studies have shown that miRNA-21 plays a significant role in cancer progression, influencing cancer cell proliferation, stemness, apoptosis, and chemoresistance [16]. Additionally, circulating miRNA-21 has the potential to serve not only as a diagnostic indicator but also as a target in cancer treatment, particularly in glioblastoma (GBM) and leptomeningeal metastasis [19,20].

While the ability to target miRNA-21 therapeutically has progressed, challenges related to cancer heterogeneity and the complexity of the tumor microenvironment has become more ostensible. Future studies will need to address the multifaceted interactions between non-coding RNAs, mRNAs, and cellular protein modulators to overcome these limitations and develop efficient RNA-based therapies for clinical application [16].

The expression of miRNA-21 in oral cancer stem cells (CSCs) has not yet been fully explored. Therefore, the objective of this study was to examine the expression levels of miR-21 in OSCC tumor tissues, isolate CSCs (CD44+ cells) from the heterogeneous tumor populations, characterize them, and compare miRNA-21 expression between CD44+ and CD44− cells. Most importantly, the study aimed to investigate the effect of miRNA-21 on CSC properties, including resistance mechanisms.

## 2. Materials and Methods

### 2.1. miRNA Network Analysis

The GEO dataset (GSE168227) was downloaded from the National Center for Biotechnology Information (NCBI) database. The dataset was selected based on its relevance to miRNA expression in oral squamous cell carcinoma (OSCC) and included sufficient miRNA expression data. Data processing and visualization were performed using Python. Differential expression analysis was conducted to compare OSCC samples with healthy controls, and miRNAs with a fold change > 2 and an adjusted *p*-value < 0.05 were considered significant. A Volcano plot was generated using the matplotlib and seaborn libraries in Python to display the log2 fold change against the −log10 *p*-value, highlighting significantly upregulated and downregulated miRNAs. The keywords “Oral squamous cell carcinoma”, “Human tissue”, and “miRNA-21”were searched in this database and the corresponding GEO datasets with enough miRNA expression information were recorded [21]. The analysis of regulatory interactions between miR-21 and key genes involved in OSCC pathogenesis was based on a review of data available in the NCBI database, i.e., from previously published studies. A table summarizing these references is included in the Supplementary Materials to support reproducibility (Table S1).

### 2.2. Patients and Tissue Samples

Tissue samples were obtained from 20 OSCC patients who were surgically treated at the Clinic for Maxillofacial Surgery of the School of Dental Medicine, University of Belgrade, and did not undergo pre-operative radio- or chemotherapy. Tumor specimens were inspected by a pathologist and squamous cell carcinoma diagnosis was established. All patients signed an informed consent. The institutional Ethical Committee approved the study, which was in agreement with the Declaration of Helsinki (approval No 36/6). The healthy gingival tissue remaining after third molar extraction served as control (n = 10). The clinicopathological status of OSCC patients is presented in Table 1.

### 2.3. Cell Cultures

The SCC-25 oral cancer cell line obtained from the American Type Culture Collection (ATCC CRL-1628™) and primary OSCC cell cultures generated by the explant culture method from five patients diagnosed with OSCC were used in the study [22]. Cells were maintained in T25 cell culture flasks in a complete medium consisting of Dulbecco’s Modified Eagle Medium (DMEM/NUT.MIX F-12 W/GLUT-I) supplemented with 10% Fetal Bovine Serum (FBS SOUTH AMERICAN), 100 U/mL penicillin-streptomycin solution (ANTIBIOTIC ANTIMYCOTIC), and 400 ng/mL hydrocortisone. Chemicals were acquired from Invitrogen, Thermo Fisher Scientific, Paisley, UK. Cells were cultured at 37 °C in a humidified atmosphere with 5% CO_2_. The complete medium was replaced every 48–72 h, and after reaching 80% confluence, CD44+ cells from the heterogeneous cell population of five primary OSCC cell cultures and the SCC-25 cell line were separated using magnetic cell sorting. For primary culture cell separation, we used cells from the second and third passage. The clinicopathological status of randomly chosen patients is given in Table S2.

### 2.4. Magnetic Cell Sorting (MACS)

MACS (Miltenyi Biotec, San Diego, CA, USA) was used to isolate CD44+ cells, as suggested by the manufacturer. In summary, adherent cells were removed using the enzymatical approach, counted, and adjusted to a concentration of 10^6^/mL. The cells were incubated with CD44 magnetic microbeads (Miltenyi Biotec, San Diego, CA, USA) for 30 min at 4 °C. The mixture was then passed through a MACS column in a magnetic separator. CD44+ cells remained in the column, while CD44− cells were collected as eluate and transferred to a new T25 flask. Once the column was removed from the magnetic field, CD44+ cells were retrieved into a separate flask.

### 2.5. Spheroid Formation Assay

Spheroids were formed by seeding CD44+ cells at a density of 10^3^ cells/mL onto 24-well culture plates pre-coated with 1 mL of poly-HEMA (poly 2-hydroxyethyl methacrylate, Sigma-Aldrich, Taufkirchen, Germany) to prevent adherence [23]. The cells were cultured in DMEM supplemented with epidermal growth factor, B-27, N2, and antibiotics (all from Sigma-Aldrich, St. Louis, MO, USA). The cultures were incubated for 3 to 10 days, after which the spheroid size was measured using ImageJ software version 1.48 (NIH, Bethesda, MD, USA; Java 1.8.9_66). To confirm the presence of CSCs in spheroid formation, the spheroids were stained with CD44−FITC antibodies and observed under a fluorescent microscope.

### 2.6. Immunocytochemistry

Cells were seeded in 6-well plates at a density of 1 × 10^4^ cells/cm^2^. Next day, cells were fixed with 4% paraformaldehyde for 20 min and incubated for 45 min at room temperature in permeabilization and blocking buffer (0.1% Triton X-100 and 10% bovine serum albumin in phosphate-buffered saline, PBS). Cells were incubated overnight at 4 °C with the CD44-APC conjugated monoclonal antibody (1:400). Nuclei were stained using 4-, 6-diamidino-2-phenylindole (DAPI, 1:4000, Molecular Probes, Eugene, OR, USA), for 10 min. After washing in PBS, cell samples were mounted with Mowiol (Sigma-Aldrich, USA) on microscope slides. Immunofluorescent images were obtained using a confocal laser scanning microscope equipped with Ar 488 and HeNe 543 and 633 laser lines (LSM 510, Carl Zeiss GmbH, Jena, Germany). Fiji-Image J software (version 1.48, NIH, Bethesda, MD, USA) was used for the micrographs analysis [24].

### 2.7. Transfection of miRNA-21 Inhibitor

CD44+ cells were seeded at a density of 1 × 10^6^ cells per well onto 6-well plates and allowed to proliferate until reaching 80% confluence; they were then transfected with miRNA21 inhibitors (MISSION Synthetic microRNA inhibitor, Human hsa-miR-21-5p) and the negative control (MISSION Synthetic microRNA inhibitor Negative Control 1) using Lipofectamine RNAiMIX (Invivogen, Termo Fisher Scientific, Waltham, MA, USA) in OptiMEM (Gibco, Grand Island, NY, USA), as recommended by the manufacturer. The control group consisted of cells to which just Lipofectamine™ RNAiMAX was added. After a period of 72 h, the inhibition of miRNA-21 was confirmed by RT-qPCR, using the TaqMan assay. Cells from the control, NC, and miRNA-21 inhibitor groups were collected for further experiments [25].

### 2.8. Transwell Cell Migration and Invasion Assay

For the migration and invasion assay, 10^5^ cells per well were seeded in the upper chamber, suspended in 500 µL of culture medium (without 10% FBS). In the lower chamber, complete medium with 10% FBS was placed as a chemo-attractant. The plate was then incubated for 24 h to allow cellular invasion. In the Transwell invasion assay, the upper chamber was first prepared by adding 30 µL of Corning^®^ Matrigel^®^ (Corning, Bedforf, MA, USA) and incubating it at 37 °C for 30 min. After incubation, non-migrated cells on the upper side of the membrane were removed using a cotton swab. The cells on the bottom side of the upper chamber membrane were fixed with a 4% paraformaldehyde solution for 30 min, followed by staining with 0.2% Crystal Violet solution (Centrohem, SP, Serbia) for another 30 min. Microscopy was used to capture images of the stained cells, subsequently analyzed by means of Fiji-ImageJ software (version 1.48; NIH, Bethesda, MD, USA).

For colorimetric quantification of the migration and invasion assay, the wells were filled with 750 μL of 10% acetic acid and incubated for 30 s with gentle shaking. This lysed the cells, releasing the Crystal Violet. The insert was then removed from the 24-well plate, and the 10% acetic acid containing the dye was transferred to a 96-well plate. A microplate reader (RT-2100C, Rayto, Shenzhen, China) was used to measure the optical density (at 540 nm) [15].

### 2.9. Apoptosis Assay Annexin V

CD44+ cells and CD44+ cells transfected with a miRNA-21 inhibitor were seeded at a density of 1 × 10^5^ cells per well in 24-well plates. After one week, apoptosis was assessed with the Annexin V–FITC Apoptosis Detection Kit (Invitrogen, Thermo Fisher Scientific, San Diego, CA, USA), following the manufacturer’s protocol. Flow cytometry (BD FACSMelody™, Franklin Lakes, NJ, USA) was applied to detect the staining, and results were displayed as dot plots comparing propidium iodide (PI) to Annexin V-FITC. The plots were divided into four regions: (a) viable cells (PI/FITC −/−; Q3), (b) early apoptotic cells (PI/FITC −/+; Q1), (c) late apoptotic cells (PI/FITC +/+; Q2), and (d) necrotic cells (PI/FITC +/−; Q4).

### 2.10. Cell Cycle Analysis

Following a 4-day period after transfection, the samples underwent centrifugation at 1400 rpm for 6 min. Subsequently, cells were rinsed with PBS for 5 min. After centrifugation, cells were resuspended in 300 µL of PBS; 700 µL of 96% cold ethanol was added slowly in little drops, and then incubated for 2 h at 4 °C. After being subjected to centrifugation (1700 rpm, 6 min) and removal of ethanol, cells were resuspended in PBS. After another round of centrifugation, the cells were resuspended in 500 µL of PBS, and 7 µL of RNase A (100 µg/mL) was added, followed by a 15-min incubation at 37 °C. A final concentration of 50 µg/mL of PI was added to the suspension and the % of cells in different cell cycle phases was established by means of flow cytometry (BD FACSMelody^TM^ Franklin Lakes, NJ, USA and BD FACSChorus^TM^ software, v3.0).

### 2.11. Isolation of RNA and Reverse-Transcription Polymerase Chain Reaction

To perform RNA extraction, cells were treated with TRIzol Reagent (Invitrogen, Thermo Fisher Scientific, UK) following the manufacturer’s instructions. RNA concentration was determined utilizing a spectrophotometer (BioSpec–nano Microvolume UV–Vis Spectrophotometer; Shimadzu Scientific Instruments, Columbia, MD, USA). Complementary DNA (cDNA) synthesis was conducted using 2 µg of total RNA with oligo d(T) primer and the RevertAid First Strand cDNA Synthesis Kit (Thermo Fisher Scientific, Waltham, MA, USA). To evaluate the miRNA levels in patients’ tissues, RNA was extracted using the same technique.

### 2.12. Real-Time Quantitative Polymerase Chain Reaction (qPCR)

A qPCR was performed with the SensiFAST SYBR Hi–ROX Kit (Bioline, London, UK), cDNA, forward and reverse primers, and water. Relative expressions of the following markers were analyzed: *OCT4*, *SOX2*, *NANOG*, *BAX*, *BCL-2*, *CASP3* (Caspase 3), *CCND1* (Cyclin D1), and *CTNNB1* (β-catenin) genes. Glyceraldehyde-3-phosphate dehydrogenase (the *GAPDH* gene), was utilized as the control gene. The 2^−ΔCT^ method was applied for the calculation of relative gene expression values [26]. The sequences of the primers utilized in this investigation are given in Table S3.

### 2.13. TaqMan microRNA Assay

Reverse transcription (RT) was performed in 15 μL reactions consisting of 100 mM dNTP, Multi Scribe Reverse Transcriptase, 3 μL of 5× concentrate miRNA-21 specific primers, RNase inhibitor, and 10× Reverse Transcription Buffer. RT conditions were: 16 °C for 30 min, 42 °C for 30 min, 85 °C for 5 min, and then cooling to 4 °C. The qPCR reaction was performed in 20 μL using a TaqMan 20× concentrate miRNA-21 (ID 000397) (Applied Biosystems, Thermo Fisher Scientific, Waltham, MA, USA), Universal PCR Master Mix, and 2 µL of the RT product. Settings for qPCR were: 50 °C for two minutes, 95 °C for 10 min, then 40 cycles of 95 °C for 15 s and 60 °C for 60 s. RNU44 (ID 001094) (Applied Biosystems, Thermo Fisher Scientific, Waltham, MA, USA) served as the miRNA internal control.

Based on the threshold cycle (Ct) value, the fold change was calculated using the formula: relative quantity (RQ) = 2^−ΔΔCT^.

### 2.14. Statistical Analysis

Statistical analysis was conducted using GraphPad Prism software version 9.0 (GraphPad Software, Inc., La Jolla, CA, USA), with results expressed as mean ± standard deviation (SD). The Kolmogorov–Smirnov test was used to assess data normality. Statistical differences between groups were determined using either Student’s *t*-test or one-way ANOVA. Statistical significance was set at * *p* < 0.05, ** *p* < 0.01, *** *p* < 0.001, and **** *p* < 0.0001. Experiments were performed in triplicate and repeated twice.

## 3. Results

### 3.1. Interaction of miRNA-21 with Key Carcinogenesis-Related Genes in OSCC

**Figure 1:**
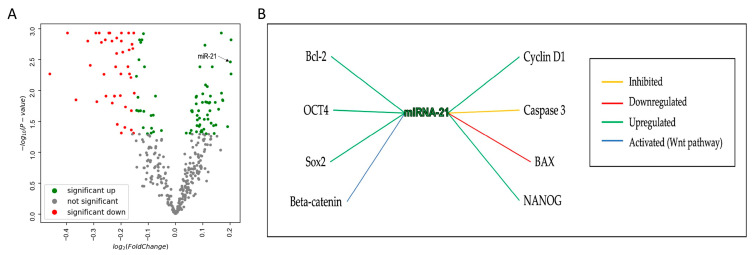
Network analysis. (**A**) Volcano plot highlights miRNA-21 in the upper-right quadrant. This position indicates that miRNA-21 is significantly upregulated in OSCC. (**B**) Visualization of the regulatory relationships between miRNA-21 and genes implicated in the pathogenesis of OSCC.

Prior to conducting in vitro assays, the GEO dataset GSE168227 (Table S4) was utilized in order to establish the significant upregulation of miRNA-21 in OSCC samples compared to healthy tissue (the dataset details are provided in the Supplementary Materials). GSE168227 identified a set of 105 miRNAs differentially expressed in OSCC, with a subset of the 19 most dysregulated miRNAs, including miRNA-21, as the microRNA signature of OSCC. miRNA-21 shows a strong upregulation in OSCC compared to healthy tissue, suggesting its potential role as an oncogenic miRNA in OSCC (Figure 1A). A review of the existing literature using the NCBI database to analyze the expression of miR-21 in OSCC and interactions between miRNA-21 and genes involved in carcinogenesis was also performed [21]. This analysis helped identify crucial interactions between the miRNA and genes involved in tumorigenesis, cell cycle control, apoptosis, and the maintenance of cancer stem cell properties. This regulatory relationship, based on previously published studies, is visualized in Figure 1B. The supporting references are summarized in a supplementary table (Table S1) [6,7,9,13,17,27,28,29,30,31]. Results showed that miRNA-21 upregulates *CCND1* (Cyclin D1), *OCT4*, *SOX2*, *NANOG*, and *BCL-2* (green lines), downregulates *BAX* (red lines), and inhibits *CASP3* (Caspase 3) (yellow line) (Figure 1B). Additionally, miRNA-21 activates *CTNNB1* (β-catenin) in the Wnt pathway (blue line).

### 3.2. The Expression Levels of miRNA-21 in OSCC Tissues

**Figure 2:**
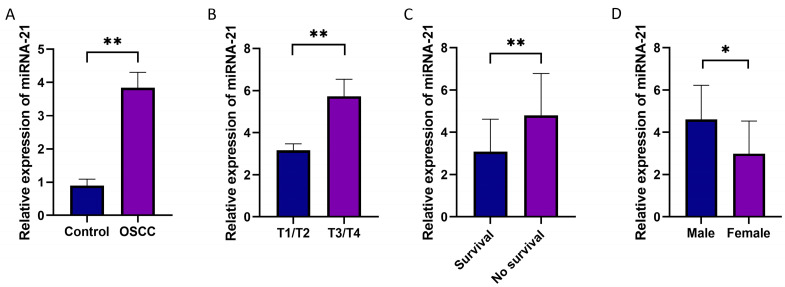
miRNA-21 expression in OSCC tissue samples. (**A**) Relative gene expression of miRNA-21 normalized to RNU44 shows significantly higher expression in OSCC (20 patients) compared to healthy controls (10 patients) (*p* < 0.01). (**B**) Stage-wise expression of miRNA-21: a significantly lower expression was found in T1/T2 (13 patients) than in T3/T4 (7 patients). (**C**) Relative gene expression of miRNA-21 in patients during 5-year survival follow-up: a significantly lower expression was found in patients that were alive (11 patients) than deceased (9 patients). (**D**) Expression of miRNA-21 in relation to gender: a higher expression was found in males (13 patients) compared to females (7 patients). * *p* < 0.05, ** *p* < 0.01.

In OSCC tissues obtained from 20 patients, higher expression (*p* = 0.003) of miRNA-21 was detected compared to healthy tissues (Figure 2A), confirming the in silico analysis. The expression of miRNA-21 was also associated with the disease stage; namely, tumors with advanced T stages (T3/4) had a significantly higher miRNA-21 expression level than the lower stages (T1/2) (*p* = 0.0015) (Figure 2B). Additionally, upregulated expression of miRNA-21 was related to lower survival (*p* = 0.01) (Figure 2C). The expression of miRNA-21 was also significantly higher in male patients compared to female patients (Figure 2D).

### 3.3. CSC Characterization

**Figure 3:**
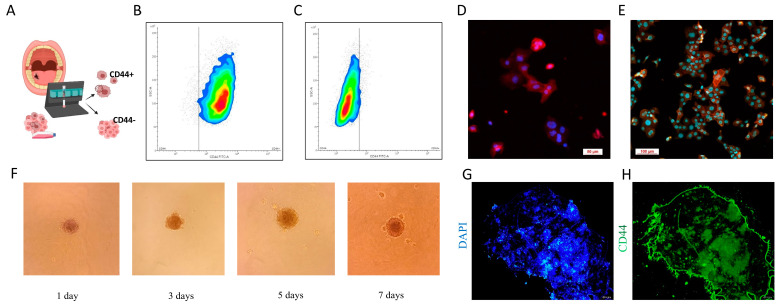
Characterization of CSCs. (**A**) The schematic illustration of magnetic separation. Flow cytometry density plots of (**B**) CD44+ and (**C**) CD44− cell populations. Confocal laser scanning microscopy images at (**D**) 40× and (**E**) 60× magnification (red fluorescence is from CD44 labelling and blue fluorescence is from DAPI). (**F**) The sphere formation assay monitored over a 7-day period, magnification 10×. Spheres stained with (**G**) DAPI and (**H**) anti-CD44 antibody were observed using a fluorescent microscope (magnification 4×).

After the successful generation of primary cell cultures, CD44+ cells were magnetically isolated. To characterize these cells, flow cytometry, immunocytochemistry, and a sphere formation assay were performed. Additionally, the expression of stemness markers *OCT4*, *SOX2*, and *NANOG* was determined using qPCR, and the results were previously published by this group [23]. The results show that in the CD44+ cell population, 95.62% of cells expressed the CD44 marker on their surface (Figure 3B), while in the CD44− cell population only 5.5% of the cells showed expression of the CD44 marker, confirming thus the purity of the CD44− population at 94.5% (Figure 3C). To visually confirm the results, confocal microscopy was performed. Cell membranes were stained red with an anti-CD44 antibody, while nuclei were stained in blue with DAPI (Figure 3D, E). Sphere formation was monitored over a 7-day period, with cells observed under a microscope, and micrographs were taken on days 1, 3, 5, and 7 (Figure 3F). The spheres were finally stained with the CD44-FITC antibody and DAPI and observed under a fluorescent microscope (Figure 3G,H).

### 3.4. CD44+ Cells Migration and Invasion

**Figure 4:**
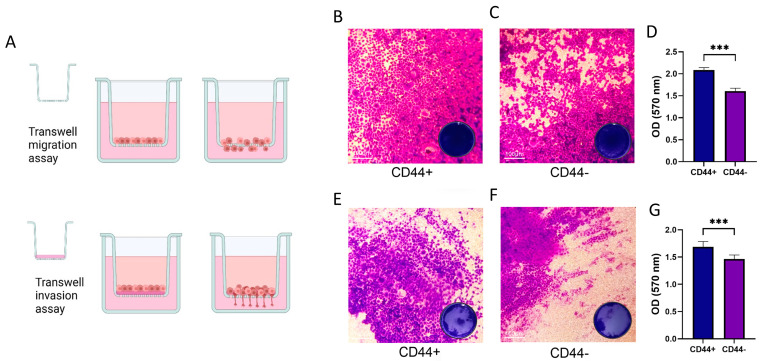
Migration and invasion assays in CD44+ and CD44− cells. (**A**) Schematic illustration of transwell assay (**B**,**C**) Transwell migration assay showing that CD44+ cells display a higher number of migrated cells compared to CD44− cells. The scale bar represents 100 μm. Magnification 10×. (**D**) Quantification of migration (optical density, OD, measured at 570 nm) confirmed that CD44+ cells have significantly higher migration ability compared to CD44− cells (*p* < 0.001). (**E**,**F**) Transwell invasion assay showed that CD44+ cells exhibit a higher invasiveness compared to CD44− cells. Magnification 10×. (**G**) Quantification indicates significantly higher invasion potential of CD44+ cells compared to CD44− cells. *** *p* < 0.001.

We aimed to compare the migration and invasion abilities of CD44+ and CD44− cell populations, and for this purpose, we used the Transwell assay, the protocol for which is schematically presented in Figure 4A. CD44+ cells exhibited a greater capacity for migration and invasion compared to CD44− cells. The quantification of staining confirmed that there was a statistically significant difference in migration (*p* = 0.0002) and invasion (*p* = 0.0001) between the two groups of cell cultures.

### 3.5. Gene Expression Profiles of CD44+ and CD44− Cells Isolated from OSCC Cultures

**Figure 5:**
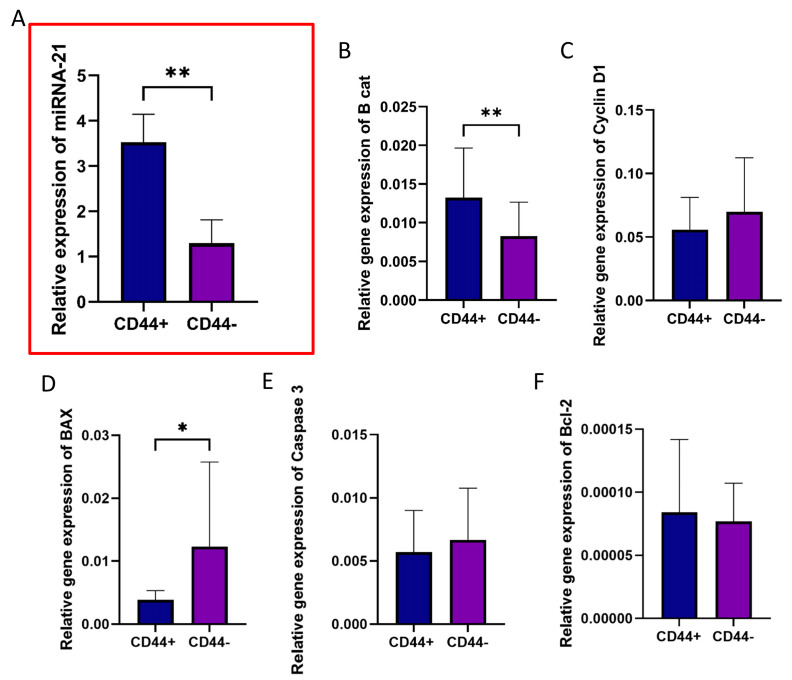
The relative gene expressions in CD44+ and CD44− cells. (**A**) miRNA-21, (**B**) *CTNNB1* (β-catenin), (**C**) *CCND1* (Cyclin D1), (**D**) *BAX*, (**E**) *CASP3* (Caspase 3), and (**F**) *BCL-2* in CD44+ and CD44− OSCC cells. * *p* < 0.05, ** *p* < 0.01.

Magnetic separation of CD44+ and CD44− OSCC cells (passage #2) was performed using the MACS system. miRNA-21 was significantly overexpressed in CD44+ cells compared to CD44− cells (*p* = 0.004) (Figure 5A). To investigate whether there is a difference between CD44+ and CD44− cells in the activity of genes related to proliferation and apoptosis, the relative gene expression of *CTNNB1* (β-catenin), *CCND1* (Cyclin D), *BAX*, *CASP3* (Caspase 3), and *BCL*-2 was determined. β-catenin, which favors CSC renewal, showed a significantly higher expression in CD44+ cells compared to CD44− cells (*p* = 0.005) (Figure 5B), while the pro-apoptotic *BAX* gene was significantly downregulated (*p* = 0.015, Figure 5D).

### 3.6. Effect of miR-21 Inhibition in CD44+ Cells

**Figure 6:**
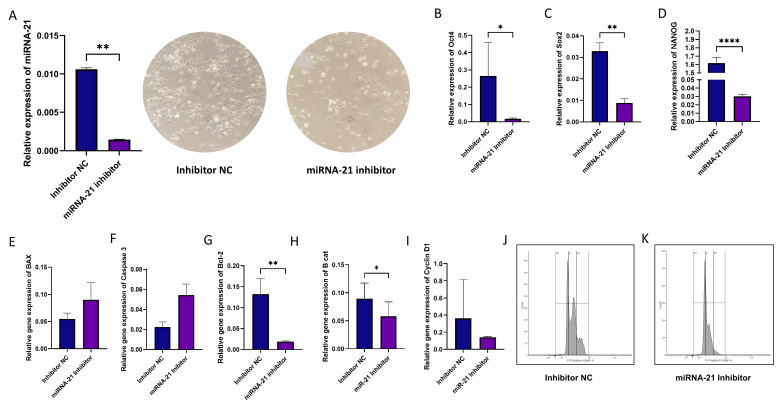
Inhibition of miR-21 in CSCs. (**A**) miRNA-21 inhibition led to a significant reduction in miRNA-21 expression levels (*p* < 0.01) and to changes in number and morphology of cells (10× magnification). (**B**–**D**) miRNA-21 inhibitor transfection effects on stemness markers; all the three analyzed cancer cell stemness markers (*OCT4*, *SOX2*, and *NANOG*) were significantly reduced. (**E**–**I**) Apoptosis, proliferation, and cell cycle markers’ expression analysis in CD44+ cells showed a highly significant reduction in *BCL-2* and β-catenin gene. (**J**,**K**) Cell cycle analysis after miRNA-21 inhibition using flow cytometry. A notable shift in the population of cells in the G0/G1 phase in miRNA-21 inhibitor-treated cells compared to the inhibitor NC group was observed, indicating an arrest in cell cycle progression. * *p* < 0.05, ** *p* < 0.01 and **** *p* < 0.0001.

Subsequent experiments were conducted solely on the CD44+ cell population. Our goal was to define the role of miRNA-21 in cancer stem cells. For this purpose, we used miRNA-21 inhibition via transfection and compared gene expression after miRNA-21 knockdown. As a control, we used a negative control (NC) for transfection inhibition. Transfection significantly reduced the levels of miRNA-21 in cells treated with the inhibitor (*p* = 0.0012) (Figure 6A). miRNA-21 inhibition was performed 72 h prior to the experiments.

We examined whether silencing miRNA-21 influenced the expression of stemness markers. Using the qPCR method, a significantly lower expression of all three examined CSC markers was detected: *OCT4* (*p* = 0.013), *SOX2* (*p* = 0.008), and *NANOG* (*p* = 0.0001), indicating reduced stemness potential in treated CSCs (Figure 6B–D). The relative gene expression of apoptosis-related genes *BAX*, *CASP 3*, *BCL-2* after transfection with the miRNA-21 inhibitor showed significant downregulation of the anti-apoptotic *BCL-2* gene (*p* = 0.008) (Figure 6G). Finally, a significant decrease in β-catenin expression (*p* < 0.05) was established as well (Figure 6H). The inhibition of miRNA-21 also affected the cell cycle, leading to cell cycle arrest in the G0/G1 phase (94.26% vs. 70.87% in cells with inhibited miR-21 and control cells, respectively).

### 3.7. Migration, Invasion, and Cell Apoptosis in CSCs Following miR-21 Inhibition

**Figure 7:**
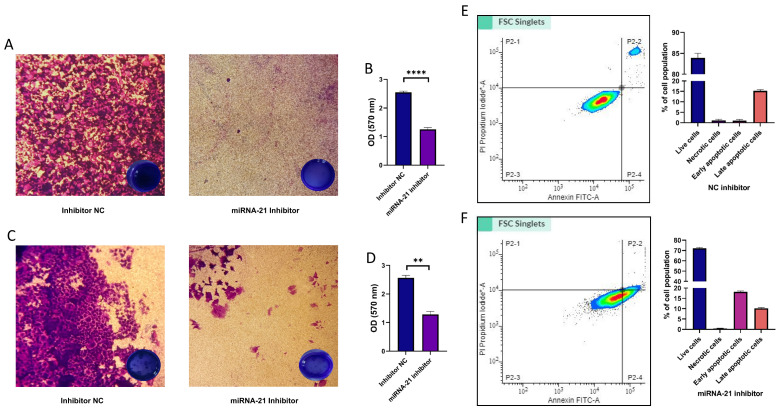
Effect of miR-21 inhibition on migration, invasion, and apoptosis of CD44+ cells. Transwell migration assay: (**A**) Representative images of Crystal Violet-stained migrating OSCC cells in control cells (left) and miRNA-21 inhibited cells (right). (**B**) Quantification of migrating cells shows a significant decrease in migration after miRNA-21 inhibition compared to the negative control. (**C**) Representative images of CD44+ invasion in controls (left) and miRNA-21 inhibited cells (right). (**D**) Quantification of the invading cells reveals a significant reduction in invasion ability in the miRNA-21 inhibitor group. Scale bar represents 100 μm. (**E**,**F**) Annexin V/FITC apoptosis assay shows the distribution of live, early apoptotic, and late apoptotic/necrotic cells in controls and miRNA-21 inhibitor-treated cells. The bar graphs display the percentage of cells in each category. miRNA-21 inhibition significantly increases the proportion of apoptotic cells. ** *p* < 0.01, and **** *p* < 0.0001.

We also examined the effect of miRNA-21 silencing on the invasion and migration of CSCs. As shown in Figure 7, inhibition significantly reduced the migration ability (*p* = 0.001), as well as the invasiveness (*p* = 0.008) of CSCs.

In addition, in order to evaluate the overall effect of expression changes in genes related to apoptosis, we performed the Annexin V assay. The results showed that miRNA-21 inhibition induced apoptosis, i.e., led to an increase in the number of cells (18.17% ± 0.4) undergoing early apoptosis.

## 4. Discussion

MicroRNAs are key regulators of various cellular functions such as development, cell division, signaling, growth, cell cycle control, maintenance of physiological balance, etc., but also contribute to cell pathology. Previous studies have shown that microRNAs play an important role in cancer initiation, progression, and therapeutic resistance, acting as oncogenes or tumor suppressors [32]. In this study, we first aimed to examine the expression levels of miRNA-21 in OSCC tissue samples and to compare them with normal tissue. The results showed that miRNA-21 levels were considerably higher in tumors than in controls and, moreover, were associated with disease stage and five-year survival rates, highlighting their potential as prognostic biomarkers in OSCC. This is in line with previous studies in which high expression of miRNA-21 in serum and tissues has been correlated with larger tumor size, metastasis, and poorer patient survival [18,33,34].

This study was driven by findings that highlight the regulatory role of miRNAs in cancer stem cells [35]. Furthermore, it has been shown that miRNAs influence critical signaling pathways such as PI3K/Akt, Wnt/β-catenin, and NF-kB, which are involved in CSC maintenance, acting as either oncogenes or tumor suppressors [7,36]. The upregulation of miR-21 in colon cancer cells, for instance, has been closely associated with an increase in CSC populations and enhanced stemness characteristics [37]. It has been reported that miRNA-21 modulates key stemness factors in pancreatic ductal adenocarcinoma, including CD44, CD133, and ALDH1, with miRNA-21 knockout significantly downregulating these factors [14]. These findings provided the rationale for focusing our research on oral cancer, aiming to understand the specific roles of miRNA-21 in OSCC CSCs.

The results from the present study offer insights into the substantial differences in gene expression profiles between CD44+ and CD44− cells isolated from OSCC. miRNA-21 was found to be significantly overexpressed in CD44+ cells compared to CD44− cells. The study also showed differential expressions of genes related to proliferation and apoptosis between CD44+ and CD44− cells. The pro-apoptotic BAX gene was significantly downregulated in CD44+ cells, indicating their lower tendency for apoptosis. β-catenin, a key component of the Wnt signaling pathway, was significantly upregulated in CD44+ cells, supporting its role in maintaining stemness and driving tumor progression. CD44+ cells have shown a higher migratory potential and invasive capability compared to the group of CD44− cells.

In pancreatic ductal adenocarcinoma (PDAC), miRNA-21 has been linked to the regulation of key stemness markers which contribute to cancer cell survival, invasion, and chemoresistance, but it may also be a therapeutic target as its knockout in PDAC cells has shown to significantly reduce cancer progression [16]. Also, miRNA-21 has been associated with glioblastoma resistance to temozolomide, the standard first-line treatment, by regulating the balance between the pro-apoptotic Bax protein and the anti-apoptotic Bcl-2, as well as by influencing Caspase 3 activity [17]. In our study, we silenced miRNA-21 specifically in OSCC CD44+ cells and examined its effects on several molecular and cellular phenotypic aspects. Consistent with the previously noted impact of miRNA-21 on stemness and survival in other cancers, we observed that silencing miRNA-21 led to a significant downregulation of key stemness markers *OCT4*, *SOX9*, and *NANOG*. It also resulted in significantly decreased expression of the anti-apoptotic gene *BCL-2*. In other words, by decreasing the levels of the anti-apoptotic marker *BCL*-2, miRNA-21 inhibition had a pro-apoptotic effect. A significant decrease in expression of the crucial player in the Wnt signaling pathway, the β-catenin gene, was established as well, suggesting on the one hand that miR-21 plays a role in Wnt/β-catenin signaling, and on the other hand that miRNA-21 silencing could reduce the self-renewal capacities of CD44+ cells [31,38]. The silencing of miRNA-21 reduced markers associated with cell survival and stemness, and induced the G0/G1 phase cell cycle arrest.

The Transwell migration assay revealed that miRNA-21 inhibition significantly reduced the migratory capacity of OSCC cells. Similarly, the invasion assay showed a significant reduction in the invasiveness of OSCC cells following miRNA-21 inhibition. Therefore, it seems that miRNA-21 plays an important role not only in facilitating migration but also in the invasive behavior of cancer cells, which is essential for metastasis. Moreover, the apoptosis assay established a major increase in the proportion of apoptotic cells after miRNA-21 knockdown. The increased apoptosis is likely tied to the observed downregulation of anti-apoptotic genes like *BCL-2*, which is fully in agreement with the findings in bladder cancer cells [30]. The present study has, however, several limitations. The number of patients included in the study and examined for microRNA levels should have been higher, with a better representation of different disease stages and grades. Moreover, since there was a difference in miRNA-21 levels between males and females, they should have also been studied separately. Kaplan–Meyer survival analysis should have been performed as well. Future studies might consider the percentage of CD44+ cells in individual tumor samples, establish a link with miRNA-21 concentrations, and correlate with clinical parameters.

## 5. Conclusions

MiRNA-21 plays a pivotal role in stemness maintenance and the regulation of CSC properties such as migration and invasion, while inhibiting apoptosis. Our results support the therapeutic potential of miRNA-21 inhibitors in reducing oral cancer cell aggressiveness and provide a strong rationale for further investigations into miRNA-21 as a biomarker and therapeutic target of CSCs in oral cancer.

## Figures and Tables

**Table 1:** Demographic and clinical characteristics of patients with OSCC.

Variables	Values (n = 20)
Gender (%)	
Male	11 (55)
Female	9 (45)
Age (years)	
Mean ± SD	58.6 ± 11.7
Min–Max	43–83
5-year survival rate	
Survival	11 (55)
No survival	9 (45)
Alcohol consumption (%)	
Yes	6 (30)
No	14 (70)
Smoking (%)	
Yes	16 (80)
No	4 (20)
T status * (%)	
T1/2	13 (65)
T3/4	7 (35)
N status ** (%)	
N_0_	16 (80)
N_1_	3 (15)
N_2_	1 (5)
M status *** (%)	
M_0_	20 (100)

* T status denotes the size and extension of the tumor: T1 cancer is ≤2 cm, not growing into nearby tissues; T2 cancer is ≥2 cm and ≤4 cm, not growing into nearby tissues; T3 cancer is ≥4 cm; in T4 the cancer is any size and is growing into nearby structures. ** N describes the spread of the tumor to regional lymph nodes: N_0_—no spread; N_1_—spread to 1 lymph node on the same side as the primary tumor but the cancer has not grown outside of the lymph node and the lymph node is ≤3 cm; N_2_—lymph node is ≥3 cm and ≤6 cm and the cancer has not grown outside of the lymph node. *** M denotes metastasis: M_0_-no distant metastasis.

## Data Availability

The original contributions presented in this study are included in the article/supplementary material. Further inquiries can be directed to the corresponding author.

## Supplementary Materials

The following supporting information can be downloaded at: https://www.mdpi.com/article/10.3390/cells14020091/s1, Table S1. Interactions between miRNA-21 and key genes involved in OSCC pathogenesis. Table S2. Clinico-pathological status of randomly chosen patients whose samples were used for the generation of primary OSCC cell cultures. Table S3. Primer Sequences used in the study. Table S4. List of micro-RNAs from the GEO database used for Figure 1A generation.
