# Supplementary material for: MicroRNA-21 as a Regulator of Cancer Stem Cell Properties in Oral Cancer
Source: Cells. 2025 Jan 10;14(2):91. doi: 10.3390/cells14020091 (PMC11763652; doi:10.3390/cells14020091)
Supplement: Supplementary file 1 [file cells-14-00091-s001.zip › cells-3394050-supplementary.pdf]

Table S1. Interactions between miRNA-21 and key genes involved in OSCC pathogenesis

GENE/PATHWAY	EFFECT OF MIR-21	REFERENCES
CASPASE 3	Inhibited	Xiao Z, 2020[28]; Chan et al., 2005[29]
BAX	Downregulated	Zhang, 2007[6]; Zhou et al., 2010[30]
CYCLIN D1	Upregulated	Yoshida, 2021[7]; Kawakita et al., 2014[27]
BCL-2	Upregulated	Xiao Z et al, 2020[28]; Shi et al., 2010[32]
B-CATENIN (WNT PATHWAY)	Activated	Jiao, Qian[9]; Kawakita et al., 2012[27]
OCT4	Upregulated	Yan ZY et al., 2017[33]
SOX2	Upregulated	Yan ZY et al., 2017[33]
NANOG	Upregulated	Bourguignon, L et al. [31]; Yan ZY et al., 2017[33];

Table S2. Clinico-pathological status of randomly chosen patients wiche samples were and used for the generation of primary OSCC cell cultures.

**Variables**<br><br>**Patients**	**Gender**	**Age**<br><br>(years)	**Localization**	**TNM status**	**Relapse**<br><br>Yes/No	**Tobacco/Alcohol consumption**<br><br>Yes/No	**5-year survival**<br><br>Yes/No
Patient 1	Female	55	Floor of the mouth	T1-2/N1/M0	No	Yes/Yes	Yes
Patient 2	Male	53	Tongue	T1-2/N0/M0	No	Yes/No	No
Patient 3	Male	58	Maxillary alveolar ridge	T4a/N0/M0	No	Yes/No	No
Patient 4	Male	51	Maxillary alveolar ridge	T4a/N0/M0	No	Yes/No	No
Patient 5	Female	70	Tongue	T3/N0/M0	No	Yes/No	Yes

Table S3. Primer Sequences used in the study.

GENE	DIRECTION	SEQUENCE
**Oct-4**	Rv	5´ TGCTCCAGCTTCTCCTTCTC 3´
	Fw	5´ GTGGAGAGCAACTCCGATG 3´
**Sox9**	Rv	5´ CTCTTTTGCACCCCTCCCATT 3´
	Fw	5´ GACTTCACATGTCCCAGCACTA 3´
**Nanog**	Rv	5´ TTTTTGCGACAGTCTTCTCTGC 3´
	Fw	5´ ATTCAGGACAGCCCTGATTCTTC 3´
**Cyclin D1**	Rv	5´GGGTGTGCAAGCCAGGTCCA 3´
	Fw	5´ CGGAGGAGAACAAACAGATC 3´
**Bcl-2**	Rv	5’ TGAGCAGAGTCTTCAGAGACAGCC 3’
	Fw	5’ ATGTGTGTGGAGAGCGTCAACC 3’
**Caspase 3**	Rv	5’ CACGCCATGTCATCATCAAC 3’
	Fw	5’ TGTTTGTGTGCTTCTGAGCC 3’
**BAX**	Rv	5’ AGTCCAATGTCCAGCCCAT 3’
	Fw	5’ ATGTTTTCTGACGGCAACTTC 3’
**β-catenin**	Rv	5’ GGTAGTGGCACCAGAATGGATT 3’
	Fw	5’ GCTACTCAAGCTGATTTGATGGA 3’
**GAPDH**	Rv	5’ CCC TGT TGC TGT AGC CAA ATT CGT 3’
	Fw	5’ TCA TGA CCA CAG TCC ATG CCA TCA 3’

Table S4. List of micro-RNAs from the GEO database used for Figure 1A generation.

ID	adj.P.Val	P.Value	t	B	logFC	ORGANISM	miRNA_ID
hsa-miR-99a	0,00117	0,00000699	-5,0178	3,6847	-0,292544	Homo sapiens	hsa-miR-99a
hsa-miR-136*	0,00117	0,00000935	-4,9332	3,416	-0,172056	Homo sapiens	hsa-miR-136*
hsa-miR-33b*	0,00117	0,00001758	4,7489	2,8354	0,168033	Homo sapiens	hsa-miR-33b*
hsa-miR-125b	0,00117	0,00001889	-4,7276	2,769	-0,280156	Homo sapiens	hsa-miR-125b
hsa-miR-30a*	0,00117	0,00001892	-4,7273	2,7678	-0,153311	Homo sapiens	hsa-miR-30a*
hsa-miR-376a	0,00117	0,00002317	-4,6674	2,5813	-0,241044	Homo sapiens	hsa-miR-376a
hsa-miR-101	0,00117	0,00002507	-4,6442	2,509	-0,244811	Homo sapiens	hsa-miR-101
hsa-miR-144	0,00117	0,00002617	-4,6315	2,4697	-0,396489	Homo sapiens	hsa-miR-144
hsa-miR-377	0,00117	0,00002852	-4,6059	2,3905	-0,198489	Homo sapiens	hsa-miR-377
hsa-miR-139-5p	0,0012	0,00003261	-4,5662	2,2676	-0,1177	Homo sapiens	hsa-miR-139-5p
hsa-miR-100	0,00141	0,00004204	-4,4904	2,0343	-0,2158	Homo sapiens	hsa-miR-100
hsa-miR-204	0,00151	0,00005208	-4,4261	1,8378	-0,123244	Homo sapiens	hsa-miR-204
hsa-miR-337-5p	0,00151	0,00006054	-4,3807	1,6998	-0,132089	Homo sapiens	hsa-miR-337-5p
hsa-miR-125a-3p	0,00151	0,00006058	4,3805	1,6992	0,203689	Homo sapiens	hsa-miR-125a-3p
hsa-miR-199b-5p	0,00151	0,00006129	-4,377	1,6886	-0,257756	Homo sapiens	hsa-miR-199b-5p
hsa-miR-143	0,00158	0,0000716	-4,33	1,5461	-0,2333	Homo sapiens	hsa-miR-143
hsa-miR-	0,0015	0,000077	-	1,478	-	Homo sapiens	hsa-miR-126*

126*	8	04	4,307 7	9	0,19892 2		
hsa-miR-451	0,0015 8	0,000077 13	- 4,307 4	1,477 9	- 0,32198 9	Homo sapiens	hsa-miR-451
hsa-miR-376c	0,0016 6	0,000085 45	- 4,276 2	1,384 1	-0,272	Homo sapiens	hsa-miR-376c
hsa-miR-29c*	0,0016 6	0,000089 78	- 4,261 2	1,338 9	- 0,12773 3	Homo sapiens	hsa-miR-29c*
hsa-miR-144*	0,0017 8	0,000101 46	- 4,223 8	1,226 9	- 0,15965 6	Homo sapiens	hsa-miR-144*
hsa-miR-602	0,0018 4	0,000109 95	4,199 2	1,153 5	0,10797 8	Homo sapiens	hsa-miR-602
hsa-miR-30e*	0,0020 9	0,000130 19	- 4,147 2	0,999	- 0,15672 2	Homo sapiens	hsa-miR-30e*
hsa-miR-497	0,0022	0,000143 14	- 4,117 9	0,912 3	- 0,17027 8	Homo sapiens	hsa-miR-497
hsa-miR-338-3p	0,0024	0,000162 83	- 4,078	0,794 6	- 0,19364 4	Homo sapiens	hsa-miR-338-3p
hsa-miR-195	0,0025 2	0,000177 79	- 4,050 7	0,714 4	- 0,21616 7	Homo sapiens	hsa-miR-195
hsa-miR-363	0,0031 7	0,000231 73	- 3,967 9	0,472 7	- 0,13423 3	Homo sapiens	hsa-miR-363
hsa-miR-21	0,0034 4	0,000260 93	3,930 5	0,364 6	0,20123 3	Homo sapiens	hsa-miR-21
hsa-miR-133a	0,0039 1	0,000306 97	- 3,879 1	0,216 7	- 0,31287 8	Homo sapiens	hsa-miR-133a
hsa-miR-145	0,0041 1	0,000334 42	- 3,852	0,138 8	- 0,17462 2	Homo sapiens	hsa-miR-145
hsa-miR-1228	0,0041 3	0,000359 67	3,828 8	0,072 6	0,13481 1	Homo sapiens	hsa-miR-1228
hsa-miR-199a-5p	0,0041 3	0,000370 55	- 3,819 3	0,045 6	- 0,21972 2	Homo sapiens	hsa-miR-199a-5p
hsa-miR-1238	0,0041 3	0,000377 15	3,813 6	0,029 5	0,08951 1	Homo sapiens	hsa-miR-1238
hsa-miR-495	0,0041 3	0,000380 74	- 3,810 6	0,020 9	- 0,11398 9	Homo sapiens	hsa-miR-495
hsa-miR-30a	0,0053 7	0,000543 14	- 3,696	- 0,301	- 0,20048	Homo sapiens	hsa-miR-30a

			3	5	9		
hsa-miR-1	0,00537	0,00054425	-3,6956	-0,3033	-0,461322	Homo sapiens	hsa-miR-1
hsa-miR-136	0,00537	0,00055068	-3,6918	-0,314	-0,1689	Homo sapiens	hsa-miR-136
hsa-miR-564	0,00537	0,00055259	3,6907	-0,3171	0,203644	Homo sapiens	hsa-miR-564
hsa-miR-126	0,00544	0,00057535	-3,6776	-0,3537	-0,262622	Homo sapiens	hsa-miR-126
hsa-miR-486-5p	0,00586	0,00063497	-3,6455	-0,443	-0,143467	Homo sapiens	hsa-miR-486-5p
hsa-miR-95	0,00616	0,00068482	-3,6208	-0,5114	-0,163811	Homo sapiens	hsa-miR-95
hsa-miR-1225-3p	0,00814	0,00092608	3,5215	-0,7841	0,110178	Homo sapiens	hsa-miR-1225-3p
hsa-miR-129-5p	0,00864	0,00100723	3,4936	-0,8599	0,116689	Homo sapiens	hsa-miR-129-5p
hsa-miR-299-5p	0,01104	0,0013367	-3,3987	-1,1147	-0,151467	Homo sapiens	hsa-miR-299-5p
hsa-miR-371-5p	0,01104	0,00134646	3,3963	-1,1213	0,168667	Homo sapiens	hsa-miR-371-5p
hsa-miR-296-5p	0,01132	0,0014113	3,3804	-1,1635	0,099889	Homo sapiens	hsa-miR-296-5p
hsa-miR-29c	0,0121	0,00154145	-3,3505	-1,2428	-0,202489	Homo sapiens	hsa-miR-29c
hsa-miR-154	0,01231	0,00160303	-3,3372	-1,278	-0,073533	Homo sapiens	hsa-miR-154
hsa-miR-30c	0,01231	0,00164669	-3,328	-1,3021	-0,224178	Homo sapiens	hsa-miR-30c
hsa-miR-30b	0,01231	0,0017019	-3,3167	-1,3317	-0,2553	Homo sapiens	hsa-miR-30b
hsa-miR-760	0,01231	0,00170408	3,3163	-1,3328	0,128022	Homo sapiens	hsa-miR-760
hsa-miR-23b	0,01231	0,0017342	-3,310	-1,348	-0,22386	Homo sapiens	hsa-miR-23b

			3	5	7		
hsa-miR-1237	0,01236	0,0017748	3,3024	-1,3693	0,077067	Homo sapiens	hsa-miR-1237
hsa-miR-425*	0,01282	0,00187593	3,2834	-1,4189	0,075389	Homo sapiens	hsa-miR-425*
hsa-miR-10b	0,01285	0,00191566	-3,2762	-1,4377	-0,137844	Homo sapiens	hsa-miR-10b
hsa-miR-133b	0,01412	0,00214369	-3,2374	-1,5384	-0,365378	Homo sapiens	hsa-miR-133b
hsa-miR-659	0,01412	0,00218075	3,2315	-1,5537	0,172589	Homo sapiens	hsa-miR-659
kshv-miR-K12-3	0,01451	0,00228074	3,2159	-1,5938	0,1737	Kaposi's sarcoma-associated herpesvirus KSHV	kshv-miR-K12-3
hsa-miR-26a	0,01513	0,002419	-3,1955	-1,6464	-0,288811	Homo sapiens	hsa-miR-26a
hsa-miR-92b	0,01524	0,00247727	3,1872	-1,6676	0,110778	Homo sapiens	hsa-miR-92b
hsa-miR-373*	0,01552	0,00268459	3,1591	-1,7394	0,145733	Homo sapiens	hsa-miR-373*
kshv-miR-K12-8	0,01552	0,00268548	3,159	-1,7397	0,1161	Kaposi's sarcoma-associated herpesvirus KSHV	kshv-miR-K12-8
hsa-miR-665	0,01552	0,00268697	3,1588	-1,7402	0,125667	Homo sapiens	hsa-miR-665
hsa-miR-939	0,01552	0,00269164	3,1582	-1,7417	0,0987	Homo sapiens	hsa-miR-939
hsa-miR-26b	0,0159	0,00283902	-3,1395	-1,7892	-0,232189	Homo sapiens	hsa-miR-26b
hsa-miR-125b-2*	0,0159	0,00284427	3,1388	-1,7909	0,123178	Homo sapiens	hsa-miR-125b-2*
hsa-miR-933	0,01637	0,0029725	3,1233	-1,8302	0,076	Homo sapiens	hsa-miR-933

hsa-miR-199b-3p	0,01848	0,00340572	-3,0751	-1,9513	-0,183711	Homo sapiens	hsa-miR-199b-3p
hsa-miR-21*	0,02018	0,00377269	3,0386	-2,0422	0,158067	Homo sapiens	hsa-miR-21*
hsa-miR-151-5p	0,02104	0,00399165	-3,0184	-2,0922	-0,142633	Homo sapiens	hsa-miR-151-5p
hsa-miR-140-5p	0,02128	0,00409875	-3,0089	-2,1157	-0,162044	Homo sapiens	hsa-miR-140-5p
hsa-miR-302c*	0,02128	0,00415159	3,0043	-2,1271	0,060722	Homo sapiens	hsa-miR-302c*
hsa-miR-191*	0,02132	0,00425148	2,9957	-2,1481	0,058822	Homo sapiens	hsa-miR-191*
hsa-miR-125a-5p	0,02132	0,00433304	-2,9889	-2,165	-0,128644	Homo sapiens	hsa-miR-125a-5p
hsa-miR-125b-1*	0,02132	0,00437565	2,9853	-2,1736	0,126156	Homo sapiens	hsa-miR-125b-1*
hsa-miR-636	0,02132	0,00439036	2,9841	-2,1766	0,084622	Homo sapiens	hsa-miR-636
hsa-miR-128	0,02151	0,00448844	-2,9761	-2,1962	-0,135544	Homo sapiens	hsa-miR-128
hsa-miR-214	0,02179	0,00460656	-2,9667	-2,2192	-0,116322	Homo sapiens	hsa-miR-214
kshv-miR-K12-9*	0,02215	0,00474194	2,9562	-2,2448	0,061411	Kaposi's sarcoma-associated herpesvirus KSHV	kshv-miR-K12-9*
hsa-miR-574-5p	0,02486	0,00538985	2,9095	-2,3579	0,117167	Homo sapiens	hsa-miR-574-5p
hsa-miR-24-1*	0,02508	0,00550537	-2,9017	-2,3766	-0,081711	Homo sapiens	hsa-miR-24-1*
hsa-miR-1234	0,02533	0,00562961	2,8935	-2,3963	0,079478	Homo sapiens	hsa-miR-1234
hsa-miR-10a	0,02543	0,00571998	-2,8877	-2,4104	-0,089689	Homo sapiens	hsa-miR-10a
hsa-miR-	0,0280	0,006385	2,847	-	0,088	Homo sapiens	hsa-miR-432

432	5	58		2,5073			
hsa-miR-1228*	0,02914	0,00671304	2,8284	-2,5513	0,119056	Homo sapiens	hsa-miR-1228*
hsa-miR-155	0,02933	0,00683573	2,8217	-2,5672	0,1055	Homo sapiens	hsa-miR-155
hsa-miR-181c*	0,02997	0,00706499	2,8094	-2,5962	0,079911	Homo sapiens	hsa-miR-181c*
hsa-miR-1226*	0,03377	0,00805268	2,7602	-2,7109	0,130544	Homo sapiens	hsa-miR-1226*
hsa-let-7f	0,0353	0,00851527	-2,7391	-2,7598	-0,213767	Homo sapiens	hsa-let-7f
hsa-miR-138-2*	0,0359	0,00875536	2,7285	-2,7841	0,089422	Homo sapiens	hsa-miR-138-2*
hsa-miR-513a-3p	0,03608	0,00889843	2,7224	-2,7983	0,148067	Homo sapiens	hsa-miR-513a-3p
hsa-miR-557	0,0369	0,0092376	2,7081	-2,831	0,139611	Homo sapiens	hsa-miR-557
hsa-miR-383	0,0369	0,00929942	2,7056	-2,8368	0,078822	Homo sapiens	hsa-miR-383
hsa-miR-324-3p	0,03795	0,00966818	2,6907	-2,8707	0,086856	Homo sapiens	hsa-miR-324-3p
hsa-miR-150*	0,03837	0,00987741	2,6825	-2,8894	0,191111	Homo sapiens	hsa-miR-150*
hsa-let-7a	0,03946	0,010267	-2,6676	-2,9231	-0,186	Homo sapiens	hsa-let-7a
hsa-miR-365	0,03988	0,01048313	-2,6596	-2,9413	-0,118444	Homo sapiens	hsa-miR-365
hsa-miR-335	0,04066	0,01079741	-2,6482	-2,967	-0,087989	Homo sapiens	hsa-miR-335
hsa-miR-615-3p	0,04224	0,0113336	2,6294	-3,0091	0,103978	Homo sapiens	hsa-miR-615-3p
hsa-let-7d	0,04344	0,01177198	-2,6147	-3,0421	-0,162322	Homo sapiens	hsa-let-7d
hsa-miR-708	0,04414	0,01208288	2,6045	-3,064	0,057544	Homo sapiens	hsa-miR-708

				7			
hsa-miR-338-5p	0,04435	0,01227726	2,5983	-3,0785	0,095333	Homo sapiens	hsa-miR-338-5p
hsa-miR-20a*	0,04435	0,01237886	-2,5951	-3,0857	-0,051744	Homo sapiens	hsa-miR-20a*
hsa-let-7b*	0,04644	0,0131605	2,5711	-3,1387	0,076889	Homo sapiens	hsa-let-7b*
hsa-miR-140-3p	0,04644	0,01337565	-2,5647	-3,1528	-0,080622	Homo sapiens	hsa-miR-140-3p
hsa-miR-320	0,04644	0,01337873	2,5646	-3,153	0,093256	Homo sapiens	hsa-miR-320
hsa-miR-17*	0,04644	0,01346746	2,562	-3,1587	0,097156	Homo sapiens	hsa-miR-17*
hsa-miR-29b	0,04863	0,01423247	-2,5402	-3,2065	-0,198944	Homo sapiens	hsa-miR-29b
hsa-miR-423-3p	0,04878	0,01448801	2,5331	-3,2218	0,039356	Homo sapiens	hsa-miR-423-3p
hsa-miR-27b	0,04878	0,01454019	-2,5317	-3,2249	-0,089456	Homo sapiens	hsa-miR-27b
hsa-miR-381	0,04886	0,01481957	-2,5241	-3,2414	-0,1542	Homo sapiens	hsa-miR-381
hsa-miR-181b	0,04886	0,01482985	2,5238	-3,242	0,053456	Homo sapiens	hsa-miR-181b
hsa-miR-520d-5p	0,04953	0,01516906	2,5148	-3,2615	0,141856	Homo sapiens	hsa-miR-520d-5p
hsa-miR-498	0,04991	0,01545073	2,5075	-3,2773	0,112278	Homo sapiens	hsa-miR-498
hsa-miR-30e	0,04991	0,01555561	-2,5048	-3,2832	-0,103156	Homo sapiens	hsa-miR-30e
hsa-let-7g	0,05095	0,01639452	-2,4838	-3,3284	-0,158333	Homo sapiens	hsa-let-7g
hsa-miR-638	0,05095	0,0164049	2,4835	-3,3289	0,121867	Homo sapiens	hsa-miR-638
hsa-let-7c	0,05095	0,01645482	-2,4823	-3,3316	-0,1328	Homo sapiens	hsa-let-7c

hsa-miR-149*	0,05095	0,0165585	2,4798	-3,337	0,088756	Homo sapiens	hsa-miR-149*
hsa-let-7f-1*	0,05095	0,01656933	2,4795	-3,3375	0,0613	Homo sapiens	hsa-let-7f-1*
hsa-miR-548d-5p	0,05096	0,01670992	2,4761	-3,3448	0,101989	Homo sapiens	hsa-miR-548d-5p
hsa-miR-30d	0,05253	0,01736743	-2,4606	-3,378	-0,098378	Homo sapiens	hsa-miR-30d
hsa-miR-361-5p	0,05257	0,01752396	-2,457	-3,3857	-0,1013	Homo sapiens	hsa-miR-361-5p
hsa-miR-548c-5p	0,05316	0,0178627	2,4492	-3,4021	0,107178	Homo sapiens	hsa-miR-548c-5p
hsa-miR-1225-5p	0,05506	0,01865902	2,4315	-3,4395	0,1522	Homo sapiens	hsa-miR-1225-5p
hsa-miR-98	0,05506	0,01879975	-2,4285	-3,4459	-0,094333	Homo sapiens	hsa-miR-98
hsa-miR-551b*	0,05519	0,01899376	2,4243	-3,4547	0,1299	Homo sapiens	hsa-miR-551b*
hsa-miR-210	0,05784	0,02006454	2,4019	-3,5017	0,085833	Homo sapiens	hsa-miR-210
ebv-miR-BART16	0,06096	0,02146252	2,3743	-3,5592	0,105911	Human herpesvirus 4	ebv-miR-BART16
hsa-miR-575	0,06096	0,02147537	2,374	-3,5597	0,129833	Homo sapiens	hsa-miR-575
hsa-miR-652	0,0617	0,0219033	-2,3659	-3,5766	-0,051011	Homo sapiens	hsa-miR-652
hcmv-miR-US4	0,06191	0,02214784	2,3613	-3,586	0,128111	Human herpesvirus 5	hcmv-miR-US4
hsa-let-7e	0,0625	0,02256613	-2,3536	-3,602	-0,121611	Homo sapiens	hsa-let-7e
hsa-miR-595	0,0625	0,02269823	2,3511	-3,6069	0,101489	Homo sapiens	hsa-miR-595
hsa-miR-532-5p	0,06437	0,02355161	-2,3358	-3,6383	-0,051644	Homo sapiens	hsa-miR-532-5p
hsa-miR-152	0,06437	0,02372449	-2,332	-3,644	-0,0693	Homo sapiens	hsa-miR-152

			8	6			
hsa-miR-134	0,06516	0,02419245	2,3246	-3,6612	0,103178	Homo sapiens	hsa-miR-134
hsa-miR-374a	0,06527	0,02441162	-2,3209	-3,6688	-0,144456	Homo sapiens	hsa-miR-374a
hsa-miR-885-3p	0,06541	0,02464001	2,317	-3,6767	0,058733	Homo sapiens	hsa-miR-885-3p
hsa-miR-20b	0,06779	0,02571928	-2,299	-3,7131	-0,117933	Homo sapiens	hsa-miR-20b
hcmv-miR-UL36	0,07042	0,02690751	2,28	-3,7514	0,082456	Human herpesvirus 5	hcmv-miR-UL36
hsa-miR-765	0,07476	0,0287681	2,2516	-3,8079	0,109822	Homo sapiens	hsa-miR-765
hsa-miR-485-3p	0,07558	0,02928841	2,244	-3,823	0,117011	Homo sapiens	hsa-miR-485-3p
hsa-miR-422a	0,07617	0,02972465	2,2377	-3,8355	0,128233	Homo sapiens	hsa-miR-422a
hsa-miR-610	0,07656	0,03008358	2,2326	-3,8456	0,076711	Homo sapiens	hsa-miR-610
hsa-miR-340	0,08063	0,03190376	-2,2074	-3,895	-0,072289	Homo sapiens	hsa-miR-340
hsa-miR-32	0,08232	0,0327944	-2,1955	-3,9182	-0,0942	Homo sapiens	hsa-miR-32
hsa-miR-378	0,08244	0,03306672	-2,1919	-3,9251	-0,096467	Homo sapiens	hsa-miR-378
hsa-miR-623	0,08317	0,03358358	2,1852	-3,9381	0,063522	Homo sapiens	hsa-miR-623
hsa-miR-186	0,08456	0,0343755	-2,1751	-3,9577	-0,089344	Homo sapiens	hsa-miR-186
hsa-miR-362-3p	0,08556	0,0350141	-2,1671	-3,9731	-0,073011	Homo sapiens	hsa-miR-362-3p
hsa-miR-29a	0,08651	0,03563646	-2,1594	-3,9878	-0,098422	Homo sapiens	hsa-miR-29a
kshv-miR-K12-10a	0,09023	0,03741431	2,1381	-4,0285	0,107311	Kaposi's sarcoma-associated	kshv-miR-K12-10a

						herpesvirus KSHV	
hsa-miR-31	0,09221	0,03848513	2,1257	-4,0521	0,165422	Homo sapiens	hsa-miR-31
hcmv-miR-UL70-3p	0,09719	0,04082668	2,0996	-4,1012	0,105433	Human herpesvirus 5	hcmv-miR-UL70-3p
hsa-miR-374b	0,10173	0,04306986	-2,0758	-4,1456	-0,106944	Homo sapiens	hsa-miR-374b
hsa-miR-138	0,10173	0,04328338	2,0736	-4,1497	0,071767	Homo sapiens	hsa-miR-138
hsa-miR-22*	0,10196	0,0436562	-2,0698	-4,1569	-0,074778	Homo sapiens	hsa-miR-22*
hsa-miR-194*	0,10656	0,04598898	2,0464	-4,1999	0,033778	Homo sapiens	hsa-miR-194*
hsa-miR-331-3p	0,10656	0,04636573	-2,0427	-4,2067	-0,0781	Homo sapiens	hsa-miR-331-3p
hsa-miR-187*	0,10656	0,0464923	2,0415	-4,2089	0,073133	Homo sapiens	hsa-miR-187*
hsa-miR-527	0,1085	0,04763572	2,0305	-4,229	0,0831	Homo sapiens	hsa-miR-527
hsa-miR-590-5p	0,11048	0,04895306	-2,0182	-4,2515	-0,058844	Homo sapiens	hsa-miR-590-5p
hsa-miR-107	0,11048	0,04937557	-2,0143	-4,2585	-0,104333	Homo sapiens	hsa-miR-107
hsa-miR-24	0,11048	0,04940198	-2,014	-4,259	-0,129744	Homo sapiens	hsa-miR-24
hsa-miR-1224-3p	0,11302	0,05084183	2,0009	-4,2826	0,092922	Homo sapiens	hsa-miR-1224-3p
ebv-miR-BART13	0,11504	0,05206209	1,9901	-4,3021	0,0966	Human herpesvirus 4	ebv-miR-BART13
hsa-miR-766	0,1179	0,05367902	1,976	-4,3272	0,083344	Homo sapiens	hsa-miR-766
hsa-miR-532-3p	0,11801	0,05404766	-1,9729	-4,3328	-0,044322	Homo sapiens	hsa-miR-532-3p
hiv1-miR-	0,12146	0,05619598	1,9549	-4,364	0,054256	Human immunodeficien	hiv1-miR-N367

N367				7		cy virus 1	
hcmv-miR-UL148D	0,12146	0,05628853	1,9541	-4,366	0,085078	Human herpesvirus 5	hcmv-miR-UL148D
hsa-miR-23a	0,12288	0,057279	-1,946	-4,3803	-0,064156	Homo sapiens	hsa-miR-23a
hsa-miR-886-3p	0,1328	0,06226343	-1,9071	-4,4482	-0,0999	Homo sapiens	hsa-miR-886-3p
hsa-miR-940	0,13536	0,06382981	1,8954	-4,4684	0,091567	Homo sapiens	hsa-miR-940
hsa-miR-16	0,13564	0,06432876	-1,8917	-4,4747	-0,091378	Homo sapiens	hsa-miR-16
hsa-miR-630	0,13782	0,06573343	-1,8815	-4,4922	-0,103511	Homo sapiens	hsa-miR-630
hsa-miR-193a-3p	0,1431	0,06871553	-1,8604	-4,528	-0,092833	Homo sapiens	hsa-miR-193a-3p
hsa-miR-769-3p	0,1431	0,0690282	1,8582	-4,5317	0,085111	Homo sapiens	hsa-miR-769-3p
hsa-miR-877*	0,14826	0,07191879	1,8386	-4,5647	0,085178	Homo sapiens	hsa-miR-877*
hsa-miR-640	0,14981	0,0732963	1,8295	-4,58	0,065067	Homo sapiens	hsa-miR-640
hsa-miR-1227	0,14981	0,07348533	1,8282	-4,5821	0,052889	Homo sapiens	hsa-miR-1227
hsa-miR-212	0,15111	0,07453253	1,8214	-4,5934	0,053544	Homo sapiens	hsa-miR-212
hsa-miR-127-3p	0,15287	0,07581259	-1,8131	-4,6071	-0,060722	Homo sapiens	hsa-miR-127-3p
hsa-miR-33a	0,15356	0,07657373	-1,8083	-4,6151	-0,056411	Homo sapiens	hsa-miR-33a
hsa-miR-30c-2*	0,15559	0,07800778	1,7993	-4,6299	0,037733	Homo sapiens	hsa-miR-30c-2*
hsa-miR-301a	0,16101	0,08115843	-1,78	-4,6615	-0,065089	Homo sapiens	hsa-miR-301a
hsa-miR-550	0,1635	0,08285583	1,7698	-4,678	0,078711	Homo sapiens	hsa-miR-550

hsa-miR-328	0,16575	0,08444746	1,7604	-4,6932	0,093022	Homo sapiens	hsa-miR-328
hsa-miR-15b	0,16792	0,08600624	-1,7514	-4,7077	-0,102622	Homo sapiens	hsa-miR-15b
hsa-miR-483-3p	0,17007	0,0877147	1,7417	-4,7233	0,089867	Homo sapiens	hsa-miR-483-3p
hsa-miR-663	0,17007	0,0881796	1,739	-4,7275	0,069656	Homo sapiens	hsa-miR-663
hsa-miR-423-5p	0,17007	0,0884934	1,7373	-4,7303	0,068844	Homo sapiens	hsa-miR-423-5p
hsa-miR-28-5p	0,17089	0,08938248	-1,7323	-4,7382	-0,074033	Homo sapiens	hsa-miR-28-5p
hsa-miR-29a*	0,17451	0,09174599	-1,7193	-4,7588	-0,026689	Homo sapiens	hsa-miR-29a*
hsa-miR-654-5p	0,17487	0,09260662	1,7146	-4,7662	0,094922	Homo sapiens	hsa-miR-654-5p
hsa-miR-1229	0,17487	0,09341709	-1,7103	-4,773	-0,063167	Homo sapiens	hsa-miR-1229
hsa-miR-379	0,17487	0,09370882	-1,7087	-4,7755	-0,043822	Homo sapiens	hsa-miR-379
hsa-miR-768-3p	0,17487	0,09383145	-1,708	-4,7765	-0,1421	Homo sapiens	hsa-miR-768-3p
hsa-miR-15a	0,17871	0,09637698	-1,6946	-4,7976	-0,077178	Homo sapiens	hsa-miR-15a
hsa-miR-20a	0,17921	0,0976449	-1,688	-4,8079	-0,114511	Homo sapiens	hsa-miR-20a
hsa-miR-103	0,17921	0,09779159	-1,6872	-4,809	-0,116044	Homo sapiens	hsa-miR-103
hcmv-miR-US25-2-5p	0,17921	0,09810262	1,6856	-4,8115	0,048544	Human herpesvirus 5	hcmv-miR-US25-2-5p
hsa-miR-188-5p	0,18373	0,10107553	1,6705	-4,8349	0,108278	Homo sapiens	hsa-miR-188-5p
hsa-miR-198	0,18432	0,10213952	1,6651	-4,843	0,093922	Homo sapiens	hsa-miR-198

				1			
hsa-miR-490-5p	0,18432	0,10240117	1,6638	-4,8451	0,070722	Homo sapiens	hsa-miR-490-5p
ebv-miR-BART4	0,18529	0,103443	1,6587	-4,853	0,059956	Human herpesvirus 4	ebv-miR-BART4
hcmv-miR-US25-1	0,18676	0,1047668	1,6522	-4,863	0,053622	Human herpesvirus 5	hcmv-miR-US25-1
hsa-miR-139-3p	0,18759	0,10574338	1,6474	-4,8702	0,065478	Homo sapiens	hsa-miR-139-3p
kshv-miR-K12-4-5p	0,19125	0,10832195	1,635	-4,8889	0,034967	Kaposi's sarcoma-associated herpesvirus KSHV	kshv-miR-K12-4-5p
hsa-miR-342-3p	0,19402	0,11041854	1,6251	-4,9038	0,0481	Homo sapiens	hsa-miR-342-3p
hsa-miR-28-3p	0,19793	0,11317845	1,6124	-4,923	0,036044	Homo sapiens	hsa-miR-28-3p
hsa-miR-877	0,19979	0,11526741	1,6028	-4,9371	0,079078	Homo sapiens	hsa-miR-877
hsa-miR-130a	0,19979	0,11532487	-1,6026	-4,9375	-0,063633	Homo sapiens	hsa-miR-130a
hsa-miR-637	0,20786	0,120548	1,5794	-4,9717	0,087289	Homo sapiens	hsa-miR-637
hsa-miR-205	0,20926	0,12192895	1,5734	-4,9804	0,119756	Homo sapiens	hsa-miR-205
hsa-miR-222	0,21047	0,12320117	1,5679	-4,9884	0,056811	Homo sapiens	hsa-miR-222
hsa-miR-124	0,21596	0,12740714	1,5502	-5,0141	0,079233	Homo sapiens	hsa-miR-124
hsa-miR-106b	0,21596	0,12759452	-1,5494	-5,0152	-0,104811	Homo sapiens	hsa-miR-106b
hsa-miR-181c	0,21596	0,12908827	-1,5432	-5,0241	-0,036889	Homo sapiens	hsa-miR-181c
hsa-miR-601	0,21596	0,12909945	1,5431	-5,0242	0,065433	Homo sapiens	hsa-miR-601
hsa-miR-483-5p	0,21596	0,12933978	1,5422	-5,025	0,084833	Homo sapiens	hsa-miR-483-5p

				6			
hsa-miR-149	0,21618	0,13006047	1,5392	-5,0298	0,043289	Homo sapiens	hsa-miR-149
hsa-miR-224	0,22235	0,13437216	1,5217	-5,0547	0,064644	Homo sapiens	hsa-miR-224
hsa-miR-298	0,22294	0,13552426	-1,5172	-5,0612	-0,037633	Homo sapiens	hsa-miR-298
hsa-miR-32*	0,22294	0,13594113	1,5155	-5,0635	0,083933	Homo sapiens	hsa-miR-32*
hsa-miR-34c-3p	0,22476	0,13765902	1,5087	-5,073	0,0357	Homo sapiens	hsa-miR-34c-3p
hsa-miR-25	0,23615	0,14527406	-1,4795	-5,1137	-0,061656	Homo sapiens	hsa-miR-25
hsa-miR-519e*	0,23709	0,14649588	1,4749	-5,12	0,055389	Homo sapiens	hsa-miR-519e*
hsa-miR-197	0,24016	0,149044	1,4655	-5,1329	0,072433	Homo sapiens	hsa-miR-197
hsa-miR-936	0,24165	0,15062259	1,4597	-5,1408	0,057167	Homo sapiens	hsa-miR-936
hsa-miR-99b	0,24594	0,15445286	-1,4459	-5,1596	-0,055989	Homo sapiens	hsa-miR-99b
hsa-miR-17	0,24594	0,15462614	-1,4452	-5,1605	-0,086411	Homo sapiens	hsa-miR-17
hsa-miR-650	0,24653	0,15567067	1,4415	-5,1655	0,040956	Homo sapiens	hsa-miR-650
hsa-miR-106a	0,25105	0,15919993	-1,4291	-5,1822	-0,060378	Homo sapiens	hsa-miR-106a
hsa-miR-185	0,25775	0,16414902	-1,412	-5,2049	-0,064478	Homo sapiens	hsa-miR-185
hsa-miR-487b	0,25956	0,16600284	-1,4057	-5,2132	-0,090767	Homo sapiens	hsa-miR-487b
hsa-miR-148b	0,26402	0,16957138	-1,3937	-5,2289	-0,043211	Homo sapiens	hsa-miR-148b
hsa-miR-1224-5p	0,26532	0,17141639	1,3876	-5,2369	0,084022	Homo sapiens	hsa-miR-1224-5p

hsa-let-7d*	0,26532	0,17184828	1,3862	-5,2388	0,062144	Homo sapiens	hsa-let-7d*
hsa-miR-425	0,27231	0,17711402	-1,369	-5,2609	-0,052689	Homo sapiens	hsa-miR-425
ebv-miR-BART19-3p	0,27337	0,17909604	-1,3627	-5,2691	-0,108044	Human herpesvirus 4	ebv-miR-BART19-3p
hsa-miR-19b	0,27337	0,17928286	-1,3621	-5,2698	-0,087811	Homo sapiens	hsa-miR-19b
hsa-miR-30c-1*	0,27454	0,18138853	1,3554	-5,2783	0,027644	Homo sapiens	hsa-miR-30c-1*
hsa-miR-887	0,27454	0,18153614	1,3549	-5,2789	0,085556	Homo sapiens	hsa-miR-887
hsa-miR-193a-5p	0,28671	0,1910003	1,3256	-5,3159	0,044456	Homo sapiens	hsa-miR-193a-5p
hsa-miR-324-5p	0,28671	0,19114273	-1,3252	-5,3164	-0,0339	Homo sapiens	hsa-miR-324-5p
hsa-miR-520d-3p	0,28945	0,19374885	1,3173	-5,3262	0,030144	Homo sapiens	hsa-miR-520d-3p
hsa-miR-141	0,2917	0,19604948	1,3104	-5,3347	0,113967	Homo sapiens	hsa-miR-141
hsa-miR-382	0,29265	0,19748063	1,3061	-5,34	0,039433	Homo sapiens	hsa-miR-382
hsa-miR-23a*	0,30093	0,20388189	1,2874	-5,3628	0,038667	Homo sapiens	hsa-miR-23a*
ebv-miR-BART12	0,30244	0,20572211	1,2821	-5,3693	0,058433	Human herpesvirus 4	ebv-miR-BART12
hsa-miR-206	0,30253	0,20660444	-1,2796	-5,3723	-0,091456	Homo sapiens	hsa-miR-206
hsa-miR-654-3p	0,30496	0,20909	-1,2725	-5,3809	-0,052256	Homo sapiens	hsa-miR-654-3p
hsa-miR-874	0,31408	0,21626693	1,2524	-5,4048	0,062633	Homo sapiens	hsa-miR-874
kshv-miR-K12-7	0,31408	0,21704493	-1,2502	-5,4074	-0,050244	Kaposi's sarcoma-associated herpesvirus	kshv-miR-K12-7

						KSHV	
hsa-miR-367	0,32023	0,22231504	-1,2358	-5,4243	-0,035633	Homo sapiens	hsa-miR-367
hsa-miR-150	0,32023	0,22303262	-1,2338	-5,4266	-0,082944	Homo sapiens	hsa-miR-150
hsa-miR-30b*	0,32225	0,22531051	1,2277	-5,4337	0,047367	Homo sapiens	hsa-miR-30b*
hsa-miR-99b*	0,32353	0,22708565	1,2229	-5,4392	0,041467	Homo sapiens	hsa-miR-99b*
hsa-miR-93	0,33072	0,23302841	-1,2072	-5,4573	-0,0671	Homo sapiens	hsa-miR-93
hsa-miR-484	0,35251	0,25098335	1,1614	-5,5087	0,0236	Homo sapiens	hsa-miR-484
hsa-miR-34a	0,35251	0,25204582	1,1588	-5,5116	0,035389	Homo sapiens	hsa-miR-34a
hsa-miR-203	0,35251	0,25217511	-1,1585	-5,5119	-0,121378	Homo sapiens	hsa-miR-203
hsa-miR-660	0,35251	0,25220064	-1,1584	-5,512	-0,029767	Homo sapiens	hsa-miR-660
hsa-miR-142-3p	0,354	0,25422988	-1,1534	-5,5175	-0,115889	Homo sapiens	hsa-miR-142-3p
hsa-miR-299-3p	0,35765	0,25782026	-1,1446	-5,5271	-0,0348	Homo sapiens	hsa-miR-299-3p
hsv1-miR-LAT	0,36914	0,26710434	1,1223	-5,5511	0,046778	Human herpesvirus 1	hsv1-miR-LAT
hsa-miR-617	0,37196	0,27014893	1,1151	-5,5588	0,040956	Homo sapiens	hsa-miR-617
hsa-miR-518c*	0,37216	0,27138909	1,1122	-5,5619	0,045189	Homo sapiens	hsa-miR-518c*
hsa-let-7i	0,37216	0,27232452	1,11	-5,5642	0,035956	Homo sapiens	hsa-let-7i
hsa-miR-181d	0,37216	0,27332376	1,1076	-5,5666	0,024578	Homo sapiens	hsa-miR-181d
hsa-miR-27a	0,38017	0,28023617	-1,091	-5,583	-0,04122	Homo sapiens	hsa-miR-27a

			6	4	2		
hsa-miR-505	0,39138	0,28955553	1,0705	-5,6052	0,021089	Homo sapiens	hsa-miR-505
hsa-miR-631	0,39173	0,29088075	1,0675	-5,6082	0,046533	Homo sapiens	hsa-miR-631
hsa-miR-500*	0,39679	0,29570987	-1,0567	-5,6191	-0,016556	Homo sapiens	hsa-miR-500*
hsa-miR-520b	0,39844	0,29802346	1,0516	-5,6242	0,048522	Homo sapiens	hsa-miR-520b
hsa-miR-542-5p	0,39864	0,29925042	1,0489	-5,6269	0,043156	Homo sapiens	hsa-miR-542-5p
hsa-miR-628-3p	0,40369	0,30413229	1,0383	-5,6375	0,028733	Homo sapiens	hsa-miR-628-3p
hsa-miR-634	0,40588	0,3068863	1,0323	-5,6434	0,017489	Homo sapiens	hsa-miR-634
hsa-miR-513a-5p	0,40638	0,3083653	1,0291	-5,6465	0,050033	Homo sapiens	hsa-miR-513a-5p
hsa-miR-885-5p	0,41555	0,31645055	1,0119	-5,6633	0,054922	Homo sapiens	hsa-miR-885-5p
hsa-miR-130b	0,41667	0,31842767	1,0077	-5,6673	0,027867	Homo sapiens	hsa-miR-130b
hsa-miR-184	0,42428	0,32539365	0,9932	-5,6812	0,040367	Homo sapiens	hsa-miR-184
hsa-miR-671-5p	0,45012	0,34643288	0,9505	-5,7209	0,042856	Homo sapiens	hsa-miR-671-5p
hsa-miR-574-3p	0,45702	0,35298316	0,9375	-5,7326	0,053644	Homo sapiens	hsa-miR-574-3p
hsa-miR-421	0,4695	0,36389144	0,9163	-5,7514	0,024456	Homo sapiens	hsa-miR-421
hsv1-miR-H1	0,47593	0,3701651	0,9043	-5,7619	0,072589	Human herpesvirus 1	hsv1-miR-H1
hsa-miR-526b	0,4905	0,38283053	0,8804	-5,7823	0,029	Homo sapiens	hsa-miR-526b
hsa-miR-572	0,49059	0,3842292	0,8778	-5,784	0,035689	Homo sapiens	hsa-miR-572

				5			
hsa-miR-19a	0,49681	0,39103793	-0,8652	-5,7951	-0,050644	Homo sapiens	hsa-miR-19a
hsa-miR-648	0,49681	0,3917957	-0,8638	-5,7962	-0,029011	Homo sapiens	hsa-miR-648
ebv-miR-BART7	0,50257	0,39769548	0,8531	-5,8051	0,036256	Human herpesvirus 4	ebv-miR-BART7
hsa-miR-223	0,50331	0,4000059	-0,8489	-5,8086	-0,059622	Homo sapiens	hsa-miR-223
hsa-miR-370	0,50331	0,40100804	-0,847	-5,81	-0,031744	Homo sapiens	hsa-miR-370
hsa-miR-200c	0,5077	0,40588251	0,8382	-5,8172	0,066744	Homo sapiens	hsa-miR-200c
hsa-miR-18b	0,51382	0,41229698	-0,8268	-5,8264	-0,020856	Homo sapiens	hsa-miR-18b
hsa-miR-362-5p	0,51382	0,41356464	-0,8245	-5,8281	-0,016589	Homo sapiens	hsa-miR-362-5p
hsa-miR-452	0,51817	0,4184659	0,8158	-5,835	0,028344	Homo sapiens	hsa-miR-452
hsa-miR-509-5p	0,52082	0,42202213	0,8096	-5,8399	0,030656	Homo sapiens	hsa-miR-509-5p
hsa-miR-662	0,52755	0,42890162	-0,7976	-5,8492	-0,0232	Homo sapiens	hsa-miR-662
hsa-miR-622	0,54306	0,44370588	0,7721	-5,8684	0,047867	Homo sapiens	hsa-miR-622
hiv1-miR-H1	0,54306	0,44445615	0,7708	-5,8694	0,036333	Human immunodeficiency virus 1	hiv1-miR-H1
hsa-miR-583	0,56757	0,46605382	0,7345	-5,8957	0,018767	Homo sapiens	hsa-miR-583
hsa-miR-200a*	0,56922	0,4700599	0,7279	-5,9003	0,014189	Homo sapiens	hsa-miR-200a*
hsa-miR-335*	0,56922	0,47049267	0,7272	-5,9008	0,0252	Homo sapiens	hsa-miR-335*
hsa-miR-151-3p	0,57763	0,47901188	-0,7132	-5,9105	-0,023833	Homo sapiens	hsa-miR-151-3p

hsa-miR-34b*	0,5788	0,48155137	-0,7091	-5,9134	-0,028144	Homo sapiens		hsa-miR-34b*
hsa-miR-513c	0,58501	0,48830425	0,6982	-5,9208	0,034711	Homo sapiens		hsa-miR-513c
hsa-miR-146a	0,59363	0,49710168	0,684	-5,9302	0,034444	Homo sapiens		hsa-miR-146a
hsa-miR-512-3p	0,61201	0,51415155	0,6571	-5,9477	0,014144	Homo sapiens		hsa-miR-512-3p
hsa-miR-492	0,64342	0,54285561	0,6127	-5,975	0,025078	Homo sapiens		hsa-miR-492
hsa-miR-10b*	0,64342	0,54402776	-0,6109	-5,976	-0,028211	Homo sapiens		hsa-miR-10b*
hsa-miR-194	0,64683	0,54866284	-0,6039	-5,9802	-0,0149	Homo sapiens		hsa-miR-194
hsa-miR-192	0,64893	0,5522055	0,5985	-5,9833	0,013289	Homo sapiens		hsa-miR-192
hsa-miR-658	0,66037	0,56372947	0,5812	-5,9932	0,011478	Homo sapiens		hsa-miR-658
hsa-miR-892b	0,67041	0,57411717	-0,5657	-6,0018	-0,016789	Homo sapiens		hsa-miR-892b
hsa-miR-455-3p	0,67709	0,58167631	-0,5545	-6,0078	-0,028422	Homo sapiens		hsa-miR-455-3p
hsa-miR-584	0,67923	0,58535209	0,5491	-6,0107	0,018233	Homo sapiens		hsa-miR-584
hsa-miR-491-3p	0,6878	0,59460019	0,5356	-6,0178	0,020444	Homo sapiens		hsa-miR-491-3p
hsa-miR-92a	0,7125	0,61789046	-0,502	-6,0347	-0,015933	Homo sapiens		hsa-miR-92a
hsa-miR-605	0,71542	0,62235761	-0,4956	-6,0378	-0,017844	Homo sapiens		hsa-miR-605
hsa-miR-208b	0,71617	0,62679163	-0,4893	-6,0408	-0,024378	Homo sapiens		hsa-miR-208b
hsa-miR-768-5p	0,71617	0,62689148	0,4891	-6,0409	0,015833	Homo sapiens		hsa-miR-768-5p
hsa-miR-	0,7390	0,648952	-	-	-	Homo sapiens		hsa-miR-409-3p

409-3p	9	79	0,458	6,0552	0,012244		
hsa-miR-566	0,75386	0,66397315	0,437	-6,0643	0,007867	Homo sapiens	hsa-miR-566
hsa-miR-770-5p	0,76269	0,67380951	0,4234	-6,07	0,012822	Homo sapiens	hsa-miR-770-5p
hsa-miR-200a	0,76891	0,68138979	0,413	-6,0743	0,030378	Homo sapiens	hsa-miR-200a
hsa-miR-454	0,78262	0,69566332	-0,3934	-6,0819	-0,009467	Homo sapiens	hsa-miR-454
hsa-miR-424	0,79013	0,70447695	0,3815	-6,0864	0,018011	Homo sapiens	hsa-miR-424
hsa-miR-513b	0,80684	0,72155987	0,3584	-6,0947	0,018389	Homo sapiens	hsa-miR-513b
hsa-miR-501-5p	0,80686	0,72376898	0,3554	-6,0958	0,007489	Homo sapiens	hsa-miR-501-5p
hsa-miR-361-3p	0,82939	0,74622507	-0,3254	-6,1057	-0,010022	Homo sapiens	hsa-miR-361-3p
hsa-miR-200b*	0,83542	0,75438019	-0,3146	-6,1091	-0,006911	Homo sapiens	hsa-miR-200b*
hsa-miR-923	0,83542	0,75617782	-0,3122	-6,1098	-0,0098	Homo sapiens	hsa-miR-923
hsa-miR-200b	0,84475	0,76691226	0,298	-6,114	0,022856	Homo sapiens	hsa-miR-200b
hsa-miR-494	0,85059	0,77452355	0,288	-6,1169	0,013822	Homo sapiens	hsa-miR-494
hsa-miR-345	0,85132	0,7774954	-0,2841	-6,118	-0,011644	Homo sapiens	hsa-miR-345
hsa-miR-10a*	0,85472	0,78291831	0,277	-6,1199	0,007767	Homo sapiens	hsa-miR-10a*
hsa-miR-215	0,86217	0,79270263	-0,2642	-6,1233	-0,008689	Homo sapiens	hsa-miR-215
hsa-miR-629*	0,86217	0,79441343	0,262	-6,1238	0,009189	Homo sapiens	hsa-miR-629*
hsa-miR-934	0,86422	0,79864027	-0,2565	-6,1252	-0,009789	Homo sapiens	hsa-miR-934

hsa-miR-339-3p	0,87606	0,81726431	0,2323	-6,131	0,006789	Homo sapiens	hsa-miR-339-3p
hsa-miR-132	0,87606	0,81787938	-0,2315	-6,1312	-0,004567	Homo sapiens	hsa-miR-132
hsa-miR-193b	0,87606	0,82012712	0,2286	-6,1318	0,007511	Homo sapiens	hsa-miR-193b
hsa-miR-801	0,87606	0,82025237	0,2284	-6,1318	0,009489	Homo sapiens	hsa-miR-801
hsa-miR-921	0,87606	0,82145184	0,2269	-6,1322	0,009767	Homo sapiens	hsa-miR-921
hsa-miR-16-2*	0,89596	0,84254507	0,1997	-6,1378	0,004111	Homo sapiens	hsa-miR-16-2*
hcmv-miR-US33-5p	0,91024	0,8604308	0,1767	-6,1421	0,004867	Human herpesvirus 5	hcmv-miR-US33-5p
hsa-miR-193b*	0,91024	0,8609679	-0,176	-6,1422	-0,005256	Homo sapiens	hsa-miR-193b*
hsa-miR-18a	0,91024	0,86349035	-0,1728	-6,1427	-0,007667	Homo sapiens	hsa-miR-18a
hcmv-miR-US33-3p	0,91024	0,86583359	0,1698	-6,1432	0,005944	Human herpesvirus 5	hcmv-miR-US33-3p
hsa-miR-671-3p	0,92292	0,88039753	0,1512	-6,1461	0,003122	Homo sapiens	hsa-miR-671-3p
hsa-miR-22	0,92591	0,88712563	0,1427	-6,1474	0,005467	Homo sapiens	hsa-miR-22
hsa-miR-493*	0,92591	0,8911205	-0,1376	-6,148	-0,004611	Homo sapiens	hsa-miR-493*
hsa-miR-135a*	0,92591	0,89204682	-0,1364	-6,1482	-0,0078	Homo sapiens	hsa-miR-135a*
hsa-miR-183*	0,92591	0,89328587	0,1348	-6,1484	0,005233	Homo sapiens	hsa-miR-183*
hsa-miR-146b-5p	0,93432	0,90393521	0,1213	-6,1501	0,007144	Homo sapiens	hsa-miR-146b-5p
hsa-miR-221	0,93626	0,9099196	0,1137	-6,151	0,005789	Homo sapiens	hsa-miR-221
hsa-miR-96	0,93626	0,91088234	0,1125	-6,151	0,006278	Homo sapiens	hsa-miR-96

				1			
hsa-let-7b	0,93995	0,91702311	-0,1047	-6,1519	-0,002867	Homo sapiens	hsa-let-7b
hsa-miR-567	0,95749	0,93673433	0,0798	-6,1542	0,001744	Homo sapiens	hsa-miR-567
hsa-miR-489	0,96891	0,9515196	0,0611	-6,1555	0,001656	Homo sapiens	hsa-miR-489
hsa-miR-181a	0,96891	0,95473016	0,0571	-6,1557	0,0022	Homo sapiens	hsa-miR-181a
hsa-miR-148a	0,96891	0,95983424	-0,0506	-6,156	-0,002978	Homo sapiens	hsa-miR-148a
hsa-miR-541	0,96891	0,96024687	-0,0501	-6,1561	-0,0013	Homo sapiens	hsa-miR-541
hsa-miR-519e	0,96891	0,96103117	0,0491	-6,1561	0,002	Homo sapiens	hsa-miR-519e
hsa-miR-142-5p	0,9781	0,97280158	0,0343	-6,1567	0,002356	Homo sapiens	hsa-miR-142-5p
hsa-miR-500	0,98113	0,97847183	-0,0271	-6,1569	-0,000744	Homo sapiens	hsa-miR-500
hsa-miR-202	0,98462	0,98462185	-0,0194	-6,1571	-0,000522	Homo sapiens	hsa-miR-202
